# Congenital Heart Disease and Pulmonary Arterial Hypertension: Current Perspectives

**DOI:** 10.31083/RCM48337

**Published:** 2026-03-20

**Authors:** Enrique Blanca-Jover, Francisco Contreras-Chova, Antonio Jerez-Calero, Jose Uberos-Fernandez, Laura Pérez-Lara

**Affiliations:** ^1^Department of Paediatrics, School of Medicine, University of Granada, 18012 Granada, Spain; ^2^Paediatric Cardiology Unit, Biohealth Research Institute Granada (Ibs. GRANADA), Clinico San Cecilio University Hospital of Granada, 18007 Granada, Spain; ^3^Paediatrics Unit, Biohealth Research Institute Granada (Ibs.GRANADA), Clinico San Cecilio University Hospital of Granada, 18007 Granada, Spain

**Keywords:** hypertension, pulmonary, heart defects, congenital, Eisenmenger complex, molecular targeted therapy, prognosis, paediatrics

## Abstract

Pulmonary arterial hypertension (PAH) is the most serious complication of congenital heart disease (CHD), constituting a heterogeneous clinical entity classified within Group 1 of the Clinical Classification of Pulmonary Hypertension (PH). PAH associated with congenital heart disease (PAH-CHD) affects approximately 3–10% of patients with CHD and accounts for up to one-third of all PAH cases in the adult population. This review provides an educational and up-to-date perspective on the epidemiology, pathophysiology, diagnosis, and management of PAH-CHD. The updated haemodynamic definitions of the 2022 European Society of Cardiology (ESC)/European Respiratory Society (ERS) guidelines (mean pulmonary artery pressure (PAP) ≥20 mmHg) and the importance of contemporary registries (COMPERA-CHD, HOPE) in defining prognosis are discussed. The pathophysiology is explored in depth, from initial shear stress to the imbalance in the three canonical pathways that regulate pulmonary vascular functions (endothelin, nitric oxide, prostacyclin), the role of inflammation and metabolism, and the central importance of the TGF-β/BMPR2 genetic pathway, which has led to new disease-modifying therapies. Moreover, this review addresses the crucial clinical distinction between paediatric management, constrained by limited evidence, and adult management (ACHD), with a focus on the multisystem disorder of Eisenmenger syndrome (ES) and the challenges of care transition. The gold-standard diagnostic (right heart catheterisation), the ‘treat and repair’ strategy in the haemodynamic ‘grey zone’, and the complex risk stratification in this population are also analysed. Additionally, the evidence from key trials (BREATHE-5, MAESTRO, REPLACE) and the paradigm shift towards initial combination therapy (AMBITION) are reviewed from a therapeutic perspective. Finally, the most significant advance is highlighted: Sotatercept, a vascular remodelling reversal agent (STELLAR study), concluding with a review of chronic complications and prospects in the field.

## 1. Introduction

Pulmonary arterial hypertension (PAH) associated with congenital heart disease (PAH-CHD) represents one of the most formidable complications in the field of modern cardiology. Defined as Group 1 of the Clinical Classification of Pulmonary Hypertension (PH), PAH results from a systemic–pulmonary shunt, which exposes the pulmonary vasculature to abnormal haemodynamic conditions [1, 2].

Historically, progression to irreversible pulmonary vascular disease (PVD), culminating in Eisenmenger syndrome (ES), was considered an inevitable outcome associated with limited survival [3, 4]. However, the landscape of PAH-CHD has been transformed by two major developments: advances in paediatric cardiac surgery and the advent of targeted therapies (TTs) for PAH.

### 1.1 The Epidemiological Burden in the Modern Era

Surgical advances have enabled more than 90% of children born with CHD to survive into adulthood [5, 6]. Consequently, this growing and ageing cohort of adults with congenital heart disease (ACHD) has changed the epidemiological profile of PAH-CHD [7]. It is estimated that this association affects between 3% and 10% of all patients with ACHD and, notably, accounts for approximately one-third of all PAH cases in the global adult population [8, 9].

Contemporary registries have been crucial in defining this population. The international COMPERA-CHD (Comparative, Prospective Registry of Newly Initiated Therapies for Pulmonary Hypertension) registry has documented that patients with PAH-CHD [7], particularly those with ES, have better long-term survival than patients with idiopathic PAH (IPAH); however, these patients also continue to experience significant morbidity [8]. Recent data from the Greek HOPE (Hellenic PulmOnary HyPertension rEgistry) have shed light on the impact of modern management strategies. The HOPE demonstrated a significant improvement in risk stratification at one year, with nearly twice as many patients achieving low-risk status at follow-up (40.9% vs. 24.7% at baseline). This change was strongly associated with the early adoption of combination therapy (73.1% of patients followed up) [9, 10]. Despite these achievements, overall survival remains a challenge; Italian data showed a 5-year survival rate of 80% in patients with PAH-CHD, which is better than that of IPAH, but remains far from optimal [1].

### 1.2 Updated Haemodynamic Definitions: The 20 mmHg Threshold

The cornerstone of diagnosing PH is invasive haemodynamic assessment via right heart catheterisation (RHC). The 2022 guidelines from the European Society of Cardiology (ESC) and the European Respiratory Society (ERS) introduced a fundamental change by lowering the evidence-based diagnostic threshold to pulmonary artery pressure mean (PAPm) ≥20 mmHg, as values above this level are associated with increased morbidity and mortality [11].

The updated haemodynamic definition of PH is now a PAPm ≥20 mmHg at rest. This definition is further subclassified to identify precapillary PAH, which is relevant in PAH-CHD. The following triad is used to define precapillary PAH:

(1). PAPm ≥20 mmHg,

(2). Pulmonary capillary wedge pressure (PCWP or pulmonary artery wedge pressure (PAWP)) ≤15 mmHg,

(3). Pulmonary vascular resistance (PVR) ≥2 Wood Units (WU).

Although 2 WU is the diagnostic threshold, it is crucial to note that guidelines and clinical trials often use a threshold of 3 WU to define ‘clinically significant’ PAH that warrants initiation of disease-targeted therapy (DTT) [1, 12].

This didactic review aims to synthesise current knowledge on PAH-CHD, focusing on molecular pathophysiology, the crucial distinction between paediatric and adult management, the evidence behind therapeutic strategies, and the impact of emerging disease-modifying therapies.

## 2. Pathophysiology: From Flow to Irreversible Remodelling

**Fig. 1.**
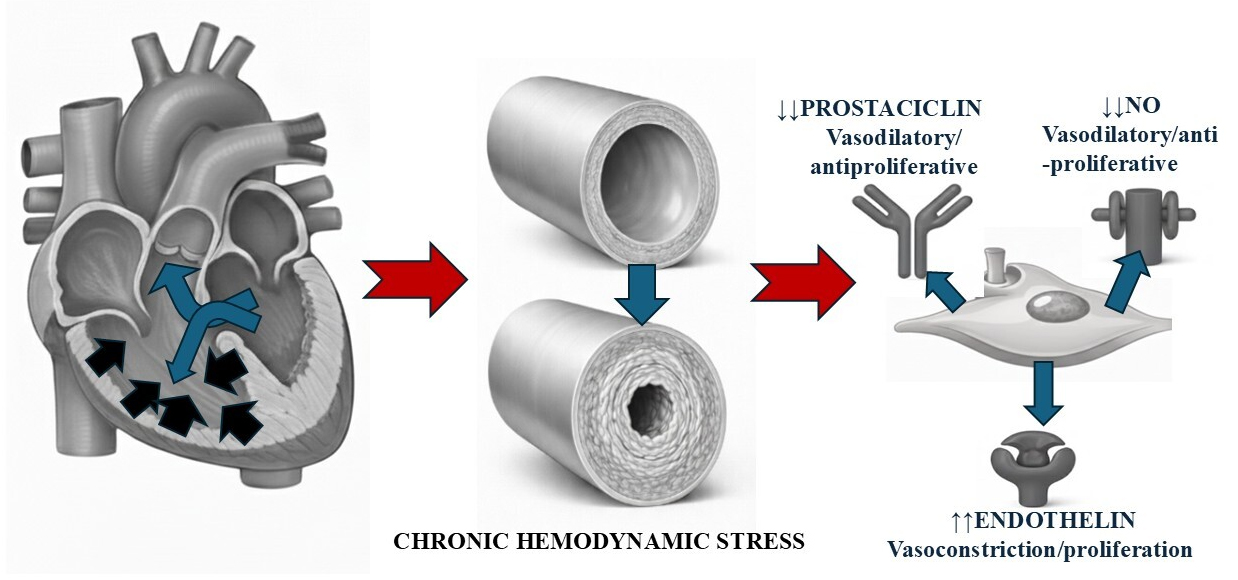
**Integrated pathophysiology of pulmonary arterial hypertension (PAH) associated with congenital heart disease with a left–right shunt, in this case caused by a ventricular septal defect (VSD)**. A schematic of the progression from an anatomical defect to vascular remodelling and molecular dysfunction. On the left, the heart shows a VSD, which causes a left-to-right shunt resulting in volume overload (blue arrows) and pressure overload of the right ventricle (hypertrophy, black arrows) and of the pulmonary circulation.. The centre panel compares a cross-section of a normal pulmonary vessel with one affected by PAH, demonstrating how chronic haemodynamic stress (shear stress and circumferential stretch) induces adverse vascular remodelling (cellular proliferation, intimal and medial thickening, and luminal narrowing). On the right, key molecular pathways underlying endothelial dysfunction are detailed, characterised by an imbalance favouring vasoconstriction and proliferation (increased endothelin) and downregulation of vasodilatory and antiproliferative pathways (decreased nitric oxide and prostacyclin signalling). NO, nitric oxide.

PAH-CHD is a clear example of how an abnormal haemodynamic stimulus can trigger a cascade of molecular dysfunction and tissue remodelling. The pathogenesis is a multiphase process that begins with mechanical stress and evolves into fixed obliterative vasculopathy (Fig. 1).

### 2.1 Initial Damage: Flow, Pressure, and Shear Stress

The primum movens of this pathology is systemic–pulmonary shunting [3, 9]. Chronic exposure of the pulmonary vascular bed, designed for a low-pressure, low-flow system, to excessive flow (pre-tricuspid shunts such as atrial septal defect, ASD) or, more aggressively, to systemic flow and pressure (post-tricuspid shunts such as ventricular septal defect, VSD, or persistent ductus arteriosus, PDA), induces chronic shear stress on pulmonary endothelial cells [13] (Table 1).

**Table 1.** **Clinical classification and characteristics of PAH-CHD (Group 1)**.

Clinical phenotype	Key characteristics	Typical shunt
Eisenmenger syndrome	Severe pulmonary vascular disease with a large, non-restrictive defect, leading to a reversed or bidirectional (R–L) shunt—results in central cyanosis.	VSD, PDA, ASD, complex congenital heart disease
PAH with systemic-to-pulmonary shunt	Moderate to large defect. PVR is elevated but does not yet exceed systemic resistance. The shunt remains predominantly L–R.	VSD, PDA, ASD
PAH with small/coincidental defects	Small defects (*e*.*g*., VSD <1 cm, ASD <2 cm) that are not considered haemodynamically significant enough to cause PAH. The clinical course is similar to that of IPAH.	Small VSD/ASD
PAH after defect correction	PAH that persists (immediate) or develops/recurs (late) years after the surgical or percutaneous closure of the defect.	Corrected VSD, ASD, PDA

PAH, pulmonary arterial hypertension; IPAH, idiopathic pulmonary hypertension; VSD, ventricular septal defect; PDA, patent ductus arteriosus; ASD, atrial septal defect; PVR, pulmonary vascular resistance; L–R, left–right; R–L, right–left.

Mechanical stress initiates endothelial dysfunction, marking a critical pathogenic turning point. Consequently, the dysfunctional endothelial cells alter the associated secretory profile, shifting from an antithrombotic and vasodilatory state to a prothrombotic, proinflammatory, and vasoconstrictive state.

### 2.2 The Molecular Axis: Imbalance in the Three Canonical Pathways

Endothelial dysfunction manifests as an imbalance in three key molecular pathways, which form the basis of all current targeted therapies (DTT) for PAH [12, 14]:

(1). Endothelin (ET-1) pathway (overactivated): ET-1 is the most potent known endogenous vasoconstrictor and a potent mitogen for vascular smooth muscle cells. In PAH-CHD, ET-1 levels are elevated, promoting vasoconstriction and cell proliferation, leading to arterial media hypertrophy. Endothelin receptor antagonists (ERAs), such as bosentan and macitentan, block this pathway.

(2). Nitric oxide (NO) pathway (deficient): NO is a crucial vasodilator produced by endothelial nitric oxide synthase (eNOS). NO acts by increasing cyclic Guanosine Monophosphate (cGMP) levels in smooth muscle cells, inducing relaxation. In PAH-CHD, NO production is decreased. Phosphodiesterase 5 inhibitors (PDE5i), such as sildenafil and tadalafil, act by blocking the degradation of cGMP, thereby enhancing NO signalling. Soluble guanylate cyclase (sGC) stimulators, such as riociguat, act synergistically by sensitising sGC to endogenous NO and, in some cases, directly stimulating the enzyme [15, 16].

(3). Prostacyclin (PGI2) pathway (deficient): PGI2 is a potent vasodilator and antiplatelet agent that acts via the cyclic Adenosine Monophosphate (cAMP) pathway. PGI2 production is reduced in PAH. Prostacyclin analogues (epoprostenol, treprostinil) and prostacyclin receptor agonists (APRAs), such as selexipag, restore signalling in this pathway [17].

This imbalance—excess ET-1 and deficiency of NO/PGI2—tilts the balance towards vasoconstriction, cell proliferation, fibrosis, and thrombosis *in situ*, laying the foundation for vascular remodelling.

### 2.3 The ‘Second Hit’: Genetics and the BMPR2/TGF-β Pathway

Although haemodynamic stress is the initiating factor, not all patients with haemodynamically similar shunts develop severe PAH. This suggests an underlying genetic susceptibility, a ‘second hit’ that modulates the response to the haemodynamic ‘first hit’. The pathway most frequently implicated is that of bone morphogenetic protein receptor type II (BMPR2), a member of the transforming growth factor beta (TGF-β) superfamily [18].

BMPR2 is a key receptor in endothelial cells that normally promotes antiproliferative, homeostatic signalling. Mutations in BMPR2 are the most common cause of hereditary PAH (HAPI) and are also found in a significant percentage of patients with PAH-CHD, suggesting a shared genetic predisposition [19].

When BMPR2 signalling is defective, the balance of the TGF-β pathway is disrupted. Antiproliferative signalling via the BMPR2/SMAD 1/5/8 pathway is reduced, while pro-proliferative signalling via the ALK-1/SMAD 2/3 pathway is increased, promoting uncontrolled growth of endothelial cells and smooth muscle, leading to obliterative remodelling [18, 20]. This insight represents one of the most important pathophysiological advances of the past decade, as it shifts the therapeutic target from simple vasodilation (the three canonical pathways) to disease modification and remodelling reversal. This is the basis for the emerging therapy with Sotatercept, a ligand trap (ActRIIA-Fc fusion protein) designed to sequester pro-proliferative ligands, such as GDF-2/BMP9, and rebalance TGF-β/BMPR2 signalling [21, 22].

### 2.4 Histopathological Findings: The Heath–Edwards Classification

The progression of PVD follows a predictable histopathological sequence, classically described by Heath and Edwards. This classification, although old, remains fundamental to understanding irreversibility:

• Grade 1: Hypertrophy of the middle layer of the pulmonary arterioles (reversible change, induced by pressure).

• Grade 2: Proliferation of the intima (cell thickening, still potentially reversible).

• Grade 3: Intimal fibrosis and progressive luminal occlusion (changes considered largely irreversible).

• Grade 4: Dilatation lesions (angiomatoid) and the formation of plexiform lesions. These are complex vascular tufts, similar to glomeruli, representing disorganised angiogenesis and are the hallmark of severe and irreversible PVD [13].

• Grades 5 and 6: Extensive fibrosis, haemosiderosis, and necrotising arteritis.

The goal of early surgical correction is to intervene before Grade 3 or higher changes become established. The goal of DTT in ES is to alleviate the effects of these already established lesions.

### 2.5 The Role of Inflammation, Thrombosis, and Metabolism

The understanding of PAH-CHD has expanded beyond the three canonical pathways and genetics. Today, it is understood as a complex syndrome in which inflammation, metabolic dysfunction, and thrombosis are active components of the disease rather than mere consequences.

• Inflammation: Endothelial dysfunction and shear stress promote the infiltration of inflammatory cells (macrophages, T/B lymphocytes, mast cells) into the pulmonary vascular wall. These cells release pro-proliferative cytokines, such as IL-6, IL-1β, and TNF-α, that perpetuate smooth muscle cell proliferation and fibrosis, creating a vicious cycle of inflammation and remodelling [12].

• Thrombosis: Endothelial dysfunction creates a pro-thrombotic state. Reduced levels of PGI2 and NO (both antiplatelet agents), together with increased von Willebrand factor (pro-thrombotic), promote *in situ* thrombosis in the pulmonary microvasculature, contributing to vascular obliteration [23].

• Metabolic dysfunction: Similar to cancer cells, proliferative cells in the vascular wall in PAH undergo a metabolic shift (the ‘Warburg effect’) towards anaerobic glycolysis, even in the presence of oxygen. This metabolic phenotype promotes resistance to apoptosis and uncontrolled cell proliferation [12].

In the specific context of PAH-CHD, these pathological processes are uniquely modulated by the chronic cyanotic environment. Unlike idiopathic PAH, patients with CHD exhibit a state of ‘metabolic inflexibility’ driven by chronic hypoxaemia. The pulmonary vasculature undergoes a metabolic shift towards glycolysis (the Warburg effect) even in the presence of oxygen, fuelling the rapid proliferation of smooth muscle cells. Furthermore, the secondary erythrocytosis typical of Eisenmenger syndrome paradoxically increases shear stress at the endothelial surface due to hyperviscosity, thereby activating pro-inflammatory and pro-thrombotic pathways. This is often compounded by systemic iron deficiency, which impairs mitochondrial function and further promotes the stabilisation of hypoxia-inducible factor (HIF)-1α, creating a self-perpetuating cycle of vascular remodelling distinct from other forms of PAH.

## 3. Critical Distinction: Paediatric versus Adult Population

PAH-CHD is not a monolithic entity. The interaction between genetic susceptibility and the type of haemodynamic shunt determines the clinical presentation, disease progression, and management, which vary dramatically depending on the age of the patient and the underlying anatomical defect.

### 3.1 The Time Factor: Anatomy of the Defect and Progression of PVD

The rate at which irreversible PVD develops depends critically on the location of the defect, which determines the nature of the haemodynamic stress:

• Post-tricuspid shunts (VSD, PDA): These defects expose the pulmonary vasculature directly to systemic pressure and high flow. Shear stress is maximal, and PVD can develop rapidly, becoming irreversible in early childhood, sometimes as early as 12–24 months of age [3, 24]. Early surgical correction is imperative.

• Pre-tricuspid shunts (ASD, partial anomalous venous drainage): These defects mainly cause volume overload in the pulmonary circulation, but at low pressure. The pulmonary vasculature can tolerate this increased flow for decades. Therefore, PAH-CHD is uncommon in these patients and typically manifests in adulthood, most often after the fourth or fifth decade of life [13]. In this setting, PAH usually involves coincidental vasculopathy or increased genetic susceptibility (*e*.*g*., BMPR2 mutation).

### 3.2 Paediatric Complexity: An ‘Orphan Disease’ Within a Rare Disease

The management of paediatric PAH is one of the greatest challenges in cardiology. PH in childhood is rare, and its aetiology is diverse, often being associated with genetic syndromes, such as Down syndrome, where PVD may progress more rapidly [18, 25, 26]. The challenges are both diagnostic and therapeutic.

#### 3.2.1 Diagnostic Challenges

• Non-specific symptoms: While adults report exertional dyspnoea, children or infants present with vague symptoms such as fatigue, irritability, diaphoresis during feeding, or failure to thrive.

• Right heart catheterisation challenges: These require deep sedation or general anaesthesia in children, which significantly alters haemodynamics (positive pressure ventilation reduces venous return, and anaesthetic agents can alter PVR), complicating the interpretation of results [27].

#### 3.2.2 Therapeutic Challenges

The main obstacle in paediatrics is the lack of robust evidence. Most pivotal clinical trials of DTTs were conducted in adults, systematically excluding children for ethical and logistical reasons (small number of patients, difficulty with endpoints such as 6-minute walk distance (6MWD)). Therefore, paediatric management is largely based on:

(1). Extrapolation of adult data: Decisions about which drug to use and at what dose are based on adult experiences and extrapolated pharmacokinetics, which is suboptimal.

(2). Data from small cohorts and surrogate markers: The available evidence comes from single-centre studies or small trials using haemodynamic markers, such as changes in PVR, rather than hard clinical outcomes (mortality, 6MWD) [27].

(3). Limited specific studies: Some data support the use of oral sildenafil [28] and inhaled treprostinil [29] in children, demonstrating short-term safety and efficacy.

Meanwhile, paediatric guidelines emphasise the importance of RHC in guiding therapy and highlight the need for management in highly specialised centres [24, 27].

The scarcity of robust data creates significant clinical dilemmas. Paediatric management remains largely empirical, relying on the off-label use of adult-approved therapies where optimal dosing—accounting for rapid developmental changes in metabolism—remains uncertain. Furthermore, standard adult endpoints, such as the 6MWD, are often infeasible or unreliable in young children due to developmental immaturity. Future research must urgently pivot away from extrapolating adult data. Priorities should include the validation of paediatric-specific multi-modal endpoints, such as actigraphy for functional assessment and cardiac magnetic resonance (CMR) for right ventricular adaptation, as well as the design of “basket trials” that group rare paediatric PH phenotypes to achieve statistical power, aiming to define whether early aggressive intervention can prevent the establishment of irreversible plexiform lesions.

### 3.3 ACHD: Physiology of the ‘Natural Survivor’

In contrast to children, adults with PAH-CHD, especially those with ES, are ‘natural survivors’. Indeed, the physiology of these adults has chronically adapted to hypoxemia and pulmonary hypertension over decades [9]. Hence, management in adults focuses less on cure, which is often no longer possible, and more on managing a complex multisystem disorder.

The key concept is right ventricular (RV) adaptation. Unlike a patient with IPAH who develops PAH in adulthood and presents with acute RV failure (an ‘unprepared’ RV), a patient with CCHD has lived with pressure overload since birth. This results in much more pronounced RV hypertrophy and adaptation, enabling preservation of systolic function and ventriculo-arterial coupling for a much longer period, despite suprasystemic PVR [30, 31, 32].

This unique adaptation explains why patients with ES have better survival than patients with HAPI, despite equivalent PVR, and why standard risk scores fail (as discussed in Section 4.4). Therefore, management of the adult focuses on addressing the consequences of this chronic adaptation:

• Haematological: Secondary erythrocytosis, iron deficiency, thrombocytopenia, coagulopathy.

• Renal: Cyanotic nephropathy, hyperuricaemia and gout.

• Musculoskeletal: Hypertrophic osteoarthropathy.

• Cerebral: Brain abscesses, strokes.

Consequently, the follow-up of these patients necessitates a structured transition from dedicated paediatric PH centres to specialised ACHD and PAH programmes, ensuring continuity of multidisciplinary expertise throughout the lifespan of the patient.

### 3.4 The Blind Spot: The Failure of the ‘Transition of Care’

A critical challenge that unites the paediatric and adult services is the ‘transition of care’. Paediatric patients with PAH-CHD, who have often been managed intensively, are frequently ‘lost’ to the healthcare system during adolescence and early adulthood. Many discontinue paediatric follow-up and do not establish care with an adult ACHD centre.

This lapse in follow-up represents a period of marked vulnerability. The AHA/ACC guidelines emphasise the need for structured transition programmes to educate patients about their disease, support treatment adherence and formally transfer care to an adult ACHD/PAH team [5, 6]. Failure to transition appropriately is a common cause of preventable clinical deterioration.

## 4. Diagnosis and Haemodynamic Stratification

The diagnosis of PAH-CHD is a stepwise process ranging from non-invasive suspicion to invasive confirmation, the latter being the final arbiter for therapeutic decision-making.

### 4.1 Non-invasive Evaluation: The Role of Transthoracic Echocardiography (TTE)

TTE is the first-line screening tool for pulmonary hypertension [1, 33]. Moreover, TTE enables estimation of pulmonary artery systolic pressure (PASP) using the maximum tricuspid regurgitation velocity (TVR max). However, in the context of CHD, TTE has significant limitations [34]. For example, in severe pulmonary stenosis, the TVR max will be high but will reflect the stenosis gradient, not PAH.

In addition to pressure estimation, the most important role of the TTE in PAH-CHD is to assess right ventricular (RV) morphology and function, which are key prognostic predictors. Signs of severe RV pressure overload include:

• RV hypertrophy and dilation.

• Flattening of the interventricular septum (D-shaped LV).

• Right atrial dilation.

• RV systolic function measurements (TAPSE, tissue S’ wave).

A composite score of four echocardiographic parameters (TAPSE <15 mm, right atrial area >25 cm^2^, RA/LA ratio >1.5, and RV systolic/diastolic duration ratio >1.5) has been shown to be strongly associated with survival in adults with ES [35].

### 4.2 The Gold Standard: Right Heart Catheterisation (RHC)

If PAH is strongly suspected, RHC is mandatory to confirm the diagnosis, classify haemodynamics and, fundamentally, determine the operability of the defect [1, 27]. Although RHC is not without risks (morbidity of 1.1%, mortality of 0.055% in expert centres), this technique remains irreplaceable [36].

In patients with shunts, RHC is technically demanding and requires:

(1). Flow measurement: Calculation of pulmonary (Qp) and systemic (Qs) flow to determine the magnitude of the shunt (Qp/Qs).

(2). Calculation of resistances: The calculation of PVR should use Qp as the denominator (PVR = (PAPm – PECP) / Qp), not systemic cardiac output (Qs), to avoid underestimating the actual resistance of the pulmonary vasculature.

(3). Acute vasoreactivity test (AVT): This test is ideally performed with inhaled nitric oxide (iNO) to determine whether high PVR is fixed or reversible.

### 4.3 Risk Stratification: A Unique Challenge in PAH-CHD

Once a diagnosis is established and the disease is considered inoperable (ES) or high risk, risk stratification is essential to guide the intensity of therapy. The ESC/ERS guidelines propose a three-tiered approach (low, intermediate, high risk) based on symptoms (WHO Functional Class), exercise capacity (6MWD), biomarkers (NT-proBNP), and haemodynamic/echocardiographic parameters (Table 2).

**Table 2.** **One-year mortality risk stratification in PAH (adapted from ESC/ERS 2022 guidelines)**.

Parameter		Low risk (<5%)	Intermediate risk (5–20%)	High risk (>20%)
Clinical exercise				
	WHO FC	I, II	III	IV
	Symptom progression	No	Slow	Rapid
	Syncope	No	Occasional	Repeated
	6MWD	>440 m	165–440 m	<165 m
Bk				
	NT-proBNP	<300 ng/L	300–1400 ng/L	>1400 ng/L
Imaging (Echo)				
	Right atrium area	<18 cm^2^	18–26 cm^2^	>26 cm^2^
	Pericardial effusion	No	Minimal	Yes
Haemodynamics				
	RAP	<8 mmHg	8–14 mmHg	>14 mmHg
	Cardiac index (CI)	>2.5 L/min/m^2^	2.0–2.4 L/min/m^2^	<2.0 L/min/m^2^
	Mixed venous O_2_ sat (SvO_2_)	>65%	60–65%	<60%

**Didactic note**: This table, designed for IPAH, must be applied with caution for PAH-CHD. ES patients are often under-stratified (*e*.*g*., ‘falsely’ better 6MWD and NT-proBNP) due to the associated unique physiology. 6MWD, 6-minute walk distance; NT-proBNP, N-terminal fragment of B-type natriuretic propeptide; WHO FC, functional class of the World Health Organisation; Bk, biomarkers; RAP, pressure in the right atrium.

However, the direct application of risk scores developed for HAPI, such as the US REVEAL 2.0 or the 4-stratum score from the ESC/ERS guidelines, to the population with PAH-CHD is problematic and may lead to a dangerous underestimation of risk [37]. The reason for this is the “natural survivor” physiology discussed above. Patients with ES, due to the associated chronic RV adaptation and a right-to-left shunt that maintains systemic cardiac output at the expense of oxygen saturation, often present with:

• Better than expected 6MWD (patients can walk further, although more desaturated).

• Paradoxically lower NT-proBNP levels than patients with PAH with equivalent PVR, as the associated RV is hypertrophied and adapted, not actively failing.

• Preserved RV systolic function (TAPSE, S’) for longer [30, 31, 32].

A patient with ES classified as “intermediate risk” according to these parameters may actually be in a far more precarious condition. Registries have identified specific predictors of mortality in PAH-CHD/ES that differ from those observed in IPAH.

Key predictors of death in ES include:

• Resting oxygen saturation (SpO2 <85%).

• Functional class (FC III or IV).

• Elevated NT-proBNP levels (although the threshold may be different).

• Renal dysfunction (cyanotic nephropathy).

• Anaemia (iron deficiency).

• Echocardiographic parameters of RV dysfunction (*e*.*g*., Moceri score) [30, 35].

Therefore, a critical need exists to use validated risk models specifically for PAH-CHD, or, if such models are unavailable, to interpret standard scores with extreme caution, placing greater weight on oxygen saturation and renal function than in HAPI [30, 37] (Table 2).

### 4.4 The Role of Biomarkers Beyond NT-proBNP

Although NT-proBNP is a pillar in the risk stratification of PAH, the usefulness of NT-proBNP in ES is, as mentioned, limited by a ‘reduced’ response from the adapted RV. Therefore, a multi-marker approach is essential in the management of ACHD. Of particular interest in this context are the following aspects:

Current risk stratification tools (*e*.*g*., REVEAL 2.0 or ESC/ERS 3-strata model) have significant limitations in PAH-CHD, as these tools do not adequately account for the unique pathophysiology of these patients. Key unmet needs in this area include:

• **Oxygen saturation**: The prognostic value of resting and exercise-induced hypoxaemia in cyanotic patients.

• **Renal and hepatic function**: The impact of chronic multi-organ congestion and hyperviscosity.

• **RV assessment**: The need for CHD-specific right ventricular function variables beyond simple TAPSE (*e*.*g*., strain or MRI-derived volumes).

## 5. Targeted Therapeutic Management: From Palliative Care to Remodelling

The management of inoperable PAH-CHD (ES) has shifted from a purely symptomatic approach to a proactive one using DTTs, based on evidence from trials in IPAH and, more recently, specific studies of PAH-CHD.

### 5.1 Treatment Goals and Escalation Strategy

The primary goal of DTTs is not merely symptom relief but modification of the disease prognosis. Modern PAH guidelines have established a clear therapeutic goal: to achieve and maintain a low-risk state [1, 14]. This state is defined by a stable patient profile that includes:

• WHO functional class (FC) I or II.

• Robust exercise capacity (*e*.*g*., 6MWD of >440 metres).

• Normal or near-normal NT-proBNP levels.

• Echocardiographic parameters showing no significant RV overload or dysfunction.

• Low-risk haemodynamics (*e*.*g*., cardiac index >2.5 L/min/m^2^, right atrial pressure <8 mmHg).

The strategy for achieving this goal is based on initial risk stratification. Patients at intermediate or high risk should start with combination therapy, and those who do not achieve low-risk status at follow-up (typically at 3–6 months) should be escalated to triple therapy, usually by adding a prostacyclin pathway drug [1, 14]. This proactive, goal-based approach has been shown to improve overall survival in PAH and is supported by data from PAH-CHD registries such as HOPE [10].

### 5.2 The Paradigm Shift: Initial Combination Therapy

Historically, treatment began with monotherapy (usually an iPDE5 or an ERA) and was only escalated after clinical deterioration (sequential therapy). This strategy has been questioned for failing to adequately treat the disease from the outset.

The AMBITION study (2015), although this study did not include patients with PAH-CHD, was a milestone in demonstrating that initial combination therapy (ambrisentan + tadalafil) was superior to monotherapy in patients with PAH (Group 1), reducing the risk of clinical failure by 50% [38].

This finding, together with registry data such as that from the HOPE study, which shows improved prognostic outcomes with combination therapy, has shifted the standard of care. Current ESC/ERS guidelines recommend initial oral combination therapy (an ERA and an iPDE5) for most intermediate-risk patients. More recent studies have even explored initial triple therapy (ERA + iPDE5 + selexipag), although the evidence of superiority over initial dual therapy remains uncertain [39, 40].

### 5.3 Specific Evidence in PAH-CC: ERAs, iPDE5, and Prostanoids

Although much of the therapy is extrapolated, there are specific pivotal trials for ES (Table 3, Ref. [17, 22, 38, 41, 42]):

**Table 3.** **Pivotal clinical trials in the management of PAH-CHD and PAH (Group 1)**.

Trial (acronym)	Drug/mechanism	Population	Key finding (endpoint)
BREATHE-5 (2006) [41]	Bosentan (dual ERA)	Eisenmenger syndrome (ES)	Seminal trial that demonstrated significant improvements in 6MWD and haemodynamics (PVR reduction). Proved DTT was safe and effective in ES.
MAESTRO (2019) [42]	Macitentan (dual ERA)	ES	Did not meet the primary 6MWD endpoint. Met key secondary endpoints (PVR reduction, NT-proBNP). Highlights that 6MWD is a poor endpoint in ES.
AMBITION (2015) [38]	Ambrisentan + Tadalafil (ERA + PDE5i)	HAPI (PAH Group 1)	Demonstrated the superiority of upfront dual combination therapy over monotherapy (50% reduction in clinical failure). Now the standard of care.
GRIPHON (2015) [17]	Selexipag (Oral Prostacyclin Receptor Agonist)	HAPI (PAH Group 1)	Demonstrated that adding an oral prostacyclin-pathway agent significantly reduced the composite morbidity and mortality endpoint.
STELLAR (2023) [22]	Sotatercept (TGF-β pathway modulator)	HAPI (PAH Group 1)	Paradigm shift. Added to background therapy, it dramatically improved 6MWD and PVR, and reduced clinical worsening by 84%. Represents disease modification.

Note: Most pivotal trials were not specifically designed for PAH-CHD; clinical recommendations are often derived from subgroup analyses and extrapolation from IPAH data. ERA, endothelin receptor antagonist; DTT, disease-targeted therapy; PDE5i, phosphodiesterase 5 inhibitors; HAPI, hereditary pulmonary arterial hypertension.

∙ **Endothelin receptor antagonists (ERAs):**

∘ BREATHE-5 (bosentan): This seminal trial (2006) demonstrated that bosentan (a dual ERA) significantly improved the primary endpoint of exercise capacity (6MWD) and haemodynamics (decreased PVR and increased cardiac index) compared with placebo in patients with SE [41, 43].

∘ MAESTRO (macitentan): This trial (2019) evaluated macitentan, a next-generation ERA, in patients with PAH. Interestingly, the study did not meet its primary endpoint of improving 6MWD at 16 weeks. However, the study did meet key secondary haemodynamic and biological endpoints, showing a significant reduction in PVR and NT-proBNP levels [42]. This discordant result is didactically important: it suggests that 6MWD is a suboptimal endpoint in patients with ES. These patients are often limited more by cyanosis (shunt) than by RV cardiac output; therefore, a DTT that improves PVR may not immediately translate into improved walking distance, even though it is improving the underlying biology (as reflected by NT-proBNP).

∙ **PDE5 inhibitors (iPDE5):**

∘ Multiple studies have demonstrated that PDE5 inhibitors (sildenafil, tadalafil) improve exercise capacity and haemodynamics in patients with ES [16, 28]. Some studies have explored the combination of ERA + PDE5 inhibitors, showing improvements in oxygen saturation and haemodynamics, although not always in 6MWD [44].

∙ **Prostacyclin pathway (prostanoids and APRAs):**

∘ Evidence for this pathway in PAH-CHD is more limited. The GRIPHON study (2015), which evaluated selexipag (an oral APRA), included a small subgroup of patients with corrected PAH-CHD, showing benefit [17, 45]. The use of selexipag in uncorrected (cyanotic) ES is more cautious due to the theoretical risk of worsening right-to-left shunting.

∘ Parenteral therapy (*e*.*g*., subcutaneous or intravenous treprostinil, epoprostenol) is reserved for the most severe and high-risk cases (CF IV), often as ‘rescue’ therapy added to dual oral therapy [46, 47]. Observational studies have shown that the addition of parenteral prostanoids in patients with SE who do not respond to oral therapy can improve haemodynamics and functional capacity [46].

### 5.4 “Treat-and-Repair” Strategy: A Bridge to Operability.

The “treat-and-repair” strategy represents the most advanced application of targeted therapy: using drugs not just for palliation but to modify the disease course sufficiently to enable a curative intervention. This approach is intended for patients in the “grey zone” (PVR 4–8 WU). The protocol involves an initial period of aggressive combination therapy (typically 6–12 months) to reverse the reversible component of vasoconstriction. Success is defined not by symptomatic improvement alone but by strict haemodynamic criteria (PVR <4 WU, Qp/Qs >1.5), confirmed by a repeat RHC, thereby allowing for safe defect closure.

**Fig. 2.**
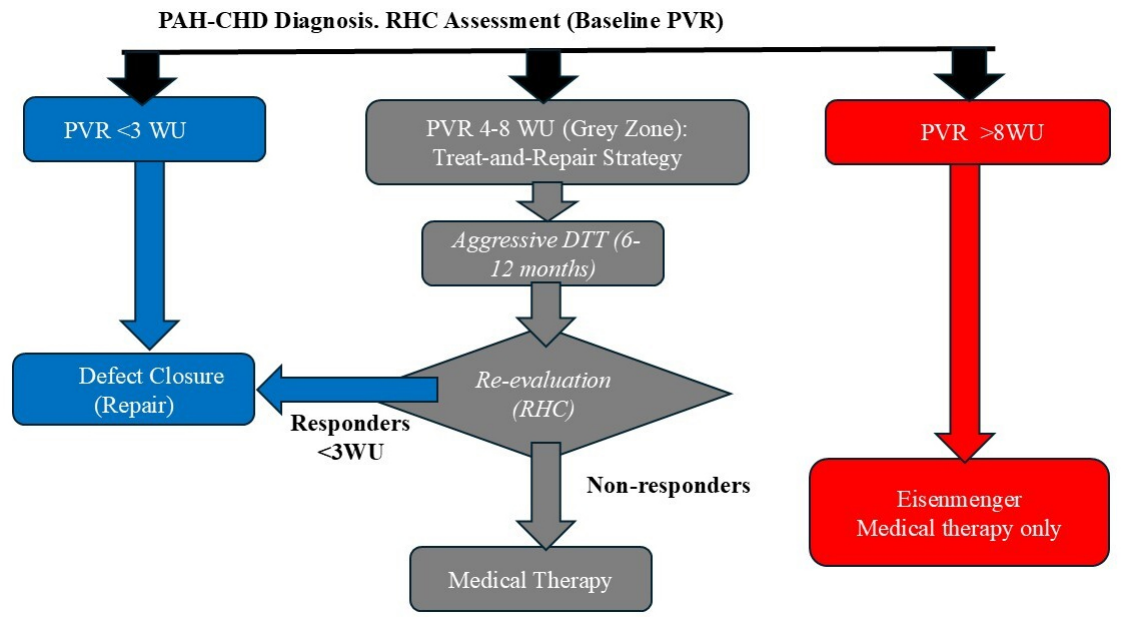
**Proposed diagnostic and therapeutic algorithm incorporating the “treat-and-repair” strategy**. This flowchart summarises contemporary clinical management for patients with PAH associated with congenital systemic-to-pulmonary shunts, such as VSD, presenting with elevated pulmonary vascular resistance (PVR) at baseline. The algorithm emphasises the critical role of comprehensive haemodynamic evaluation. The “treat-and-repair” strategy entails initiating specific pulmonary vascular-targeted drug therapy (often upfront combination therapy) to reverse vascular remodelling and lower PVR. Haemodynamic re-evaluation after pharmacological treatment is indicated to identify patients who have achieved established criteria for operability, subsequently permitting surgical or percutaneous closure of the defect (repair) with reduced perioperative risk. RHC, right heart catheterisation; PVR, pulmonary vascular resistance; DTT, disease-targeted therapy; WU, Wood units.

The RHC stratifies patients into three categories that dictate management (Fig. 2):

(1). **Operable (low PVR):** Generally defined by a PVR <4 WU^2^. These patients should be referred for defect closure (surgical or percutaneous) [3, 28].

(2). **Inoperable (ES):** Defined by a PVR >8 WU^2^ or a PVR that is a very high fraction of systemic resistance (PVR/systemic vascular resistance (SVR) >0.5–0.75). In these patients, the shunt acts as a safety valve for the RV. Closure of the defect is absolutely contraindicated, as it would eliminate this safety valve, causing acute RV failure and death [4, 13]. Management is palliative with a DTT.

(3). **The “grey zone” (borderline PVR):** Defined by a PVR between 4 and 8 WU^2^, these patients represent the greatest clinical challenge. Closure of the defect is high risk because it may lead to early or late postoperative PAH.

For patients in this “grey zone”, a “treat-and-repair” strategy has been established [13, 28], representing a fundamental change in management that avoids high-risk defect closure. This strategy involves:

• Initiating DTTs (usually a combination therapy) for PAH.

• Waiting several months (6–12) to allow the DTT to reduce the PVR.

• Performing a second RHC to reassess haemodynamics.

• If PVR has decreased significantly (*e*.*g*., <4 WU^2^) and AVT is favourable, low-risk closure of the defect may be considered.

### 5.5 Therapeutic Failures and the Logic of Combination: The REPLACE Trial

This therapeutic strategy is not without failures. Importantly, ‘more’ is not always ‘better’. The RE-PLACE trial (2021) challenged the common practice of switching from an iPDE5 to riociguat (sGC) in patients who did not respond adequately. The study did not demonstrate the superiority of switching to riociguat over continuing iPDE5 therapy (both in combination with an ERA). In fact, there were more treatment discontinuations due to hypotension in the riociguat group [48]. This suggests that once the NO/cGMP pathway is being treated, switching agents within the same pathway may not offer additional benefits and may increase side effects. These findings support the rationale for multi-pathway combination therapy (acting on different pathways) rather than sequential monotherapy or switching agents.

### 5.6 The Paradigm Shift: Disease-Modifying Therapies (Sotatercept)

The development of sotatercept represents the most significant advance in PAH therapy in a decade. As discussed in the pathophysiology section, this drug is not a vasodilator. It is a fusion protein (an ActRIIA-Fc trap ligand) that rebalances *BMPR2/TGF-β* signalling, promotes antiproliferative signalling, and actively reverses vascular remodelling [18, 21].

The Phase 3 STELLAR trial (2023) evaluated sotatercept added to optimised baseline therapy (most patients were already on triple therapy) in patients with PAH, including a subgroup of corrected PAH-CC. The results were compelling:

∙ The primary endpoint, change in 6MWD at 24 weeks, improved by 40.8 metres in the sotatercept group [22].

∙ The sotatercept group showed dramatic improvements in 8 of 9 secondary endpoints, including a 40% mean reduction in PVR and an 84% reduction in the risk of death or clinical deterioration [22].

Sotatercept represents a paradigm shift from vasodilator palliation to biological modification of the disease. However, the role of sotatercept in uncorrected (ES) PAH-CHD remains to be defined; nonetheless, sotatercept treatment opens the possibility of ‘reversing’ PVR in the future.

While sotatercept represents a paradigm shift, integrating this therapy into the PAH-CHD algorithm requires careful clinical stratification. In patients with uncorrected ES, a specific concern is the potential of the drug to increase haemoglobin levels and cause telangiectasias. Given that ES patients already face a precarious balance between secondary erythrocytosis, thrombocytopenia, and a bleeding diathesis, the safety profile regarding thrombotic and haemorrhagic events must be rigorously evaluated in this specific sub-population. A key unanswered question remains its optimal sequencing: should it be reserved for patients who have failed triple therapy, or introduced earlier to exploit its anti-remodelling potential? Ongoing trials such as ZENITH and HYPERION will be pivotal in determining if reversing vascular remodelling can reopen a window for operability (“treat-and-repair”) in defects previously deemed inoperable.

### 5.7 Supportive Therapies

The management of PAH-CHD is not limited to DTTs—supportive therapies are essential for managing symptoms and improving quality of life:

∙**Diuretics**: Essential for managing volume overload and right heart failure.

∙**Oxygen therapy**: Although controversial in PAH (since hypoxaemia is due to shunting rather than lung disease), oxygen therapy is used to improve saturation at rest, during exercise, or during air travel.

∙**Rehabilitation and exercise**: Historically discouraged, supervised physical training (rehabilitation) has been shown to be safe and effective in patients with stable PAH, improving exercise capacity and quality of life [49].

## 6. Management of Chronic Complications in ES

The management of patients with ES is an exercise in internal medicine and palliative care, focused on anticipating and treating the sequelae of chronic hypoxaemia.

### 6.1 Haematological Complications: The Balance Between Thrombosis and Haemorrhage

∙**A. Secondary erythrocytosis:** Chronic hypoxaemia stimulates erythropoietin, leading to polycythaemia. Unlike polycythaemia vera, this erythrocytosis is an adaptive response. However, when excessive (Hct >65%), this response can lead to hyperviscosity syndrome (headache, dizziness, blurred vision).

∘ Management: Routine phlebotomy is strongly contraindicated [9]. Repeated phlebotomy induces iron deficiency, which results in microcytic red cells that increase blood viscosity at a given haematocrit, paradoxically increasing the risk of stroke. Phlebotomy is reserved only for acute symptoms of hyperviscosity and should always be followed by volume replacement (isovolaemic) [3].

∙**B. Iron deficiency:** This is common (due to microbleeds) and deleterious. Iron deficiency, even without anaemia, is associated with poorer functional capacity and survival [50]. Careful treatment with oral iron supplements is essential.

∙**C. The anticoagulation paradigm:** Thrombosis vs. haemorrhage: Patients with ES live in a haematological paradox. These patients have a high risk of *in situ* pulmonary artery thrombosis (20–30% of patients) due to flow stasis in dilated pulmonary arteries, endothelial dysfunction, and hyperviscosity [23]. Meanwhile, these patients are also at high risk of haemorrhage (thrombocytopenia, impaired platelet function, fragile bronchial collaterals).

∘ Management: Routine anticoagulation is not recommended. This treatment option is reserved for patients with clear indications: atrial fibrillation, previous paradoxical embolism, or confirmed extensive pulmonary thrombi [3, 51]. The choice of agent is complex. Data on direct oral anticoagulants (DOACs) in ACHD are limited; studies such as that by Freisinger *et al*. [52] suggest that the use of DOACs is increasing, but the safety of these agents in patients with cyanotic shunts (especially right-to-left) remains unestablished. Complex anatomy, renal dysfunction, and drug interactions mean that expert centres must individualise management with warfarin or DOACs.

### 6.2 Cardiovascular and Pulmonary Complications

∙ Left coronary artery compression (LCA): In severe PAH, the pulmonary artery (PA) can dilate massively (diameter >40 mm), compressing the left main coronary artery between the PA and the aorta. This can cause angina, ischaemia, or sudden death. Diagnosis is made by computed tomography (CT) angiography, and treatment is usually LCA stenting [53, 54].

∙ Haemoptysis: This is a feared and potentially fatal complication. Haemoptysis may be due to rupture of fragile bronchial collateral vessels that receive flow from the high-pressure systemic circulation, or, less commonly, to pulmonary thrombosis/infarction. Bronchial artery embolisation is used to manage massive haemoptysis.

∙ Arrhythmias: Atrial fibrillation and flutter are common due to extreme dilatation of the right atrium from chronic pressure overload. These conditions have a very poor prognosis, increase the risk of thrombosis, and require anticoagulation [4].

### 6.3 Systemic Complications (Neurological, Renal, and Gout)

∙**Neurological events:** Patients with ES are at high risk for central nervous system (CNS) complications.

∘ Cerebrovascular accident (CVA): This may be thrombotic (due to hyperviscosity, atrial fibrillation) or, more commonly, a paradoxical embolism. Any thrombus formed in the venous system (*e*.*g*., deep vein thrombosis) can cross the right-to-left shunt (VSD, ASD) and lodge in the cerebral circulation.

∘ Cerebral abscess: The liver normally filters bacteria from the portal system. In the presence of an R–L shunt, systemic venous blood, which may contain transient bacteria, bypasses both the pulmonary and hepatic filters, potentially seeding the brain and causing abscesses, a complication associated with high mortality [4].

∙**Cyanotic nephropathy and gout:** Chronic hypoxaemia and erythrocytosis induce renal haemodynamic changes (hyperflow, glomerular hyperfiltration). Over time, this leads to focal and segmental glomerulosclerosis, resulting in cyanotic nephropathy. Clinically, this manifests as proteinuria and eventually chronic renal failure, which is an independent predictor of poor prognosis [4]. In addition, hyperviscosity and high cell turnover in erythrocytosis increase uricacid production, while decreased renal function reduces its excretion, leading to hyperuricaemia and secondary gout.

∙**Hypertrophic osteoarthropathy:** Chronic hypoxaemia induces proliferation in the periosteum in long bones, causing significant bone and joint pain (acropachy or ‘clubbing’ of the fingers is the most visible manifestation) [4].

### 6.4 Pregnancy: An Absolute Contraindication

Pregnancy poses the greatest risk for a woman with ES. The physiological changes of pregnancy (increased intravascular volume, dilutional anaemia, a hypercoagulable state, and, critically, a fall in SVR) are catastrophic. The drop in SVR worsens the right-to-left shunt, deepening cyanosis. The highest risk of death (30–50% maternal mortality) occurs during delivery or the postpartum period, due to haemodynamic collapse from blood loss and the sudden increase in SVR as the uterus contracts [55, 56].

In addition to the immediate risks, pregnancy should be viewed as a physiological ‘stress test’ for the cardiovascular system and a critical prognostic window for identifying high-risk young women. Information on pregnancy-related history (*e*.*g*., hypertensive disorders or gestational diabetes) remains essential for long-term cardiovascular risk stratification, even in women with repaired or less advanced disease.

∘ Management: Pregnancy is contraindicated [1, 4, 55]. Rigorous contraceptive counselling and the use of long-acting reversible contraception are imperative. It is important to note that drugs such as ERAs and riociguatare are teratogenic [15].

## 7. Transplantation and Future Prospects

### 7.1 Transplantation as a Last Resort

For patients with ES and PAH-CHD who progress to RV failure refractory to maximum DTTs, transplantation is the only remaining therapeutic option [5, 9]. The criteria for referral to the transplant list are those for high-risk PAH: persistent CF III-IV, 6MWD <300 m, recurrent syncope, diuretic–refractory RV failure, or development of massive haemoptysis or LCA compression.

The choice of procedure is complex:

(1). **Heart–lung transplantation:** This was the historical standard, as it replaces both failed systems.

(2). **Bilateral lung transplantation + defect repair:** This is currently the preferred strategy. Hypertrophied RV can often recover remarkably once faced with a low-resistance pulmonary vascular bed. This strategy preserves the native heart and optimises the allocation of donor organs [57, 58].

Post-transplant survival in ES is comparable to or superior to that in HAPI, with reported 1-, 5-, and 10-year survival rates of approximately 73%, 51%, and 28%, respectively [3, 58].

### 7.2 Future Directions: Towards Precision Medicine

The future of PAH-CHD management focuses on shifting from a reactive to a proactive and personalised approach:

(1). **Specific risk models**: There is an urgent need to develop and validate risk scores (similar to REVEAL [37]) that are specifically designed for the unique physiology of PAH-CC, incorporating RV function and biomarkers.

(2). **Reducing dependence on RHC:** RHC is invasive. The development of more robust non-invasive tools (3D ECHO, RV strain, cardiac magnetic resonance imaging to measure flow and PVR) is key for long-term follow-up [33, 34, 59].

(3). **Additional remodelling therapies:** In addition to sotatercept, other disease-modifying pathways targeting inflammation, metabolism, and proliferation are being investigated, such as tyrosine kinase inhibitors (seralutinib) and other modulators of metabolic and epigenetic pathways [12, 18, 48].

(4). **Artificial intelligence (AI): **The use of deep learning to integrate multimodal data (clinical, genetic, imaging, haemodynamic) will enable the prediction of disease trajectories and the identification of optimal therapeutic windows before clinical deterioration.

## 8. Conclusions

PAH-CHD has evolved from a uniformly fatal condition to a manageable, albeit complex and multisystemic, chronic disease. Updated haemodynamic definitions (mPAP ≥20 mmHg) may allow for earlier identification, while registries such as COMPERA and HOPE confirm that targeted therapy, especially initial combination therapy, has tangibly improved prognosis.

Management remains a didactic challenge, with a significant gap between the paediatric population, where evidence is scarce, and the adult population (ACHD), where the focus is on the multi-organ sequelae of ES. The cornerstone of diagnosis remains RHC, which is essential for defining operability and guiding the “treat and repair” strategy in borderline cases.

The future is promising. The success of sotatercept in the STELLAR trial heralds a paradigm shift away from mere palliative vasodilation toward active disease modification and reversal of vascular remodelling. As research advances towards precision medicine, the clinical management of PAH-CHD continues to require a multidisciplinary and highly specialised approach to navigate this unique and challenging physiology.

## References

[1] Humbert M, Kovacs G, Hoeper MM, Badagliacca R, Berger RMF, Brida M (2022). 2022 ESC/ERS Guidelines for the diagnosis and treatment of pulmonary hypertension. *European Heart Journal*.

[2] Simonneau G, Montani D, Celermajer DS, Denton CP, Gatzoulis MA, Krowka M (2019). Haemodynamic definitions and updated clinical classification of pulmonary hypertension. *The European Respiratory Journal*.

[3] Jone PN, Ivy DD, Hauck A, Karamlou T, Truong U, Coleman RD (2023). Pulmonary Hypertension in Congenital Heart Disease: A Scientific Statement From the American Heart Association. *Circulation. Heart Failure*.

[4] Arvanitaki A, Gatzoulis MA, Opotowsky AR, Khairy P, Dimopoulos K, Diller GP (2022). Eisenmenger Syndrome: JACC State-of-the-Art Review. *Journal of the American College of Cardiology*.

[5] Stout KK, Daniels CJ, Aboulhosn JA, Bozkurt B, Broberg CS, Colman JM (2019). 2018 AHA/ACC Guideline for the Management of Adults With Congenital Heart Disease: Executive Summary: A Report of the American College of Cardiology/American Heart Association Task Force on Clinical Practice Guidelines. *Journal of the American College of Cardiology*.

[6] Baumgartner H, De Backer J, Babu-Narayan SV, Budts W, Chessa M, Diller GP (2021). 2020 ESC Guidelines for the management of adult congenital heart disease. *European Heart Journal*.

[7] Diller GP, Gatzoulis MA (2007). Pulmonary vascular disease in adults with congenital heart disease. *Circulation*.

[8] Kaemmerer H, Gorenflo M, Huscher D, Pittrow D, Apitz C, Baumgartner H (2020). Pulmonary Hypertension in Adults with Congenital Heart Disease: Real-World Data from the International COMPERA-CHD Registry. *Journal of Clinical Medicine*.

[9] Patsiou V, Arvanitaki A, Farmakis IT, Anthi A, Demerouti E, Apostolopoulou S (2025). Survival prospects of pulmonary arterial hypertension associated with congenital heart disease: Insights from the HOPE registry. *International Journal of Cardiology*.

[10] Arvanitaki A, Boutsikou M, Anthi A, Apostolopoulou S, Avgeropoulou A, Demerouti E (2019). Epidemiology and initial management of pulmonary arterial hypertension: real-world data from the Hellenic pulmOnary hyPertension rEgistry (HOPE). *Pulmonary Circulation*.

[11] Manes A, Palazzini M, Leci E, Bacchi Reggiani ML, Branzi A, Galiè N (2014). Current era survival of patients with pulmonary arterial hypertension associated with congenital heart disease: a comparison between clinical subgroups. *European Heart Journal*.

[12] Ruopp NF, Cockrill BA (2022). Diagnosis and Treatment of Pulmonary Arterial Hypertension: A Review. *JAMA*.

[13] Ferrero P, Constantine A, Chessa M, Dimopoulos K (2024). Pulmonary arterial hypertension related to congenital heart disease with a left-to-right shunt: phenotypic spectrum and approach to management. *Frontiers in Cardiovascular Medicine*.

[14] Kylhammar D, Kjellström B, Hjalmarsson C, Jansson K, Nisell M, Söderberg S (2018). A comprehensive risk stratification at early follow-up determines prognosis in pulmonary arterial hypertension. *European Heart Journal*.

[15] Ghofrani HA, Galiè N, Grimminger F, Grünig E, Humbert M, Jing ZC (2013). Riociguat for the treatment of pulmonary arterial hypertension. *The New England Journal of Medicine*.

[16] Galiè N, Brundage BH, Ghofrani HA, Oudiz RJ, Simonneau G, Safdar Z (2009). Tadalafil therapy for pulmonary arterial hypertension. *Circulation*.

[17] Sitbon O, Channick R, Chin KM, Frey A, Gaine S, Galiè N (2015). Selexipag for the Treatment of Pulmonary Arterial Hypertension. *The New England Journal of Medicine*.

[18] Rosenzweig EB, Abman SH, Adatia I, Beghetti M, Bonnet D, Haworth S (2019). Paediatric pulmonary arterial hypertension: updates on definition, classification, diagnostics and management. *The European Respiratory Journal*.

[19] Zhao Q, Zhang R, Shi J, Xie H, Zhang L, Li F (2023). Imaging Features in BMPR2 Mutation-associated Pulmonary Arterial Hypertension. *Radiology*.

[20] Galambos C, Mullen MP, Shieh JT, Schwerk N, Kielt MJ, Ullmann N (2019). Phenotype characterisation of TBX4 mutation and deletion carriers with neonatal and paediatric pulmonary hypertension. *The European Respiratory Journal*.

[21] Moceri P, Kempny A, Liodakis E, Alonso Gonzales R, Germanakis I, Diller GP (2015). Physiological differences between various types of Eisenmenger syndrome and relation to outcome. *International Journal of Cardiology*.

[22] Hoeper MM, Badesch DB, Ghofrani HA, Gibbs JSR, Gomberg-Maitland M, McLaughlin VV (2023). Phase 3 Trial of Sotatercept for Treatment of Pulmonary Arterial Hypertension. *The New England Journal of Medicine*.

[23] Broberg CS, Ujita M, Prasad S, Li W, Rubens M, Bax BE (2007). Pulmonary arterial thrombosis in eisenmenger syndrome is associated with biventricular dysfunction and decreased pulmonary flow velocity. *Journal of the American College of Cardiology*.

[24] Lopes AA, Barst RJ, Haworth SG, Rabinovitch M, Al Dabbagh M, Del Cerro MJ (2014). Repair of congenital heart disease with associated pulmonary hypertension in children: what are the minimal investigative procedures? Consensus statement from the Congenital Heart Disease and Pediatric Task Forces, Pulmonary Vascular Research Institute (PVRI). *Pulmonary Circulation*.

[25] Hopper RK, Abman SH, Elia EG, Avitabile CM, Yung D, Mullen MP (2023). Pulmonary Hypertension in Children with Down Syndrome: Results from the Pediatric Pulmonary Hypertension Network Registry. *The Journal of Pediatrics*.

[26] Diller GP, Körten MA, Bauer UMM, Miera O, Tutarel O, Kaemmerer H (2016). Current therapy and outcome of Eisenmenger syndrome: data of the German National Register for congenital heart defects. *European Heart Journal*.

[27] Del Cerro MJ, Moledina S, Haworth SG, Ivy D, Al Dabbagh M, Banjar H (2016). Cardiac catheterization in children with pulmonary hypertensive vascular disease: consensus statement from the Pulmonary Vascular Research Institute, Pediatric and Congenital Heart Disease Task Forces. *Pulmonary Circulation*.

[28] Singh TP, Rohit M, Grover A, Malhotra S, Vijayvergiya R (2006). A randomized, placebo-controlled, double-blind, crossover study to evaluate the efficacy of oral sildenafil therapy in severe pulmonary artery hypertension. *American Heart Journal*.

[29] Krishnan U, Takatsuki S, Ivy DD, Kerstein J, Calderbank M, Coleman E (2012). Effectiveness and safety of inhaled treprostinil for the treatment of pulmonary arterial hypertension in children. *The American Journal of Cardiology*.

[30] Kempny A, Hjortshøj CS, Gu H, Li W, Opotowsky AR, Landzberg MJ (2017). Predictors of Death in Contemporary Adult Patients With Eisenmenger Syndrome: A Multicenter Study. *Circulation*.

[31] Hopkins WE, Waggoner AD (2002). Severe pulmonary hypertension without right ventricular failure: the unique hearts of patients with Eisenmenger syndrome. *The American Journal of Cardiology*.

[32] Hopkins WE (2005). The remarkable right ventricle of patients with Eisenmenger syndrome. *Coronary Artery Disease*.

[33] Rudski LG, Lai WW, Afilalo J, Hua L, Handschumacher MD, Chandrasekaran K (2010). Guidelines for the echocardiographic assessment of the right heart in adults: a report from the American Society of Echocardiography endorsed by the European Association of Echocardiography, a registered branch of the European Society of Cardiology, and the Canadian Society of Echocardiography. *Journal of the American Society of Echocardiography: Official Publication of the American Society of Echocardiography*.

[34] D’Alto M, Bossone E, Opotowsky AR, Ghio S, Rudski LG, Naeije R (2018). Strengths and weaknesses of echocardiography for the diagnosis of pulmonary hypertension. *International Journal of Cardiology*.

[35] Moceri P, Dimopoulos K, Liodakis E, Germanakis I, Kempny A, Diller GP (2012). Echocardiographic predictors of outcome in eisenmenger syndrome. *Circulation*.

[36] Hoeper MM, Lee SH, Voswinckel R, Palazzini M, Jais X, Marinelli A (2006). Complications of right heart catheterization procedures in patients with pulmonary hypertension in experienced centers. *Journal of the American College of Cardiology*.

[37] Benza RL, Gomberg-Maitland M, Elliott CG, Farber HW, Foreman AJ, Frost AE (2019). Predicting Survival in Patients With Pulmonary Arterial Hypertension: The REVEAL Risk Score Calculator 2.0 and Comparison With ESC/ERS-Based Risk Assessment Strategies. *Chest*.

[38] Hoeper MM, McLaughlin VV, Barberá JA, Frost AE, Ghofrani HA, Peacock AJ (2016). Initial combination therapy with ambrisentan and tadalafil and mortality in patients with pulmonary arterial hypertension: a secondary analysis of the results from the randomised, controlled AMBITION study. *The Lancet. Respiratory Medicine*.

[39] Chin KM, Sitbon O, Doelberg M, Feldman J, Gibbs JSR, Grünig E (2021). Three- Versus Two-Drug Therapy for Patients With Newly Diagnosed Pulmonary Arterial Hypertension. *Journal of the American College of Cardiology*.

[40] White RJ, Jerjes-Sanchez C, Bohns Meyer GM, Pulido T, Sepulveda P, Wang KY (2020). Combination Therapy with Oral Treprostinil for Pulmonary Arterial Hypertension. A Double-Blind Placebo-controlled Clinical Trial. *American Journal of Respiratory and Critical Care Medicine*.

[41] Galiè N, Beghetti M, Gatzoulis MA, Granton J, Berger RMF, Lauer A (2006). Bosentan therapy in patients with Eisenmenger syndrome: a multicenter, double-blind, randomized, placebo-controlled study. *Circulation*.

[42] Gatzoulis MA, Landzberg M, Beghetti M, Berger RM, Efficace M, Gesang S (2019). Evaluation of Macitentan in Patients With Eisenmenger Syndrome. *Circulation*.

[43] Diller GP, Dimopoulos K, Kaya MG, Harries C, Uebing A, Li W (2007). Long-term safety, tolerability and efficacy of bosentan in adults with pulmonary arterial hypertension associated with congenital heart disease. *Heart (British Cardiac Society)*.

[44] D’Alto M, Romeo E, Argiento P, Sarubbi B, Santoro G, Grimaldi N (2012). Bosentan-sildenafil association in patients with congenital heart disease-related pulmonary arterial hypertension and Eisenmenger physiology. *International Journal of Cardiology*.

[45] Beghetti M, Channick RN, Chin KM, Di Scala L, Gaine S, Ghofrani HA (2019). Selexipag treatment for pulmonary arterial hypertension associated with congenital heart disease after defect correction: insights from the randomised controlled GRIPHON study. *European Journal of Heart Failure*.

[46] D’Alto M, Constantine A, Balint OH, Romeo E, Argiento P, Ablonczy L (2019). The effects of parenteral prostacyclin therapy as add-on treatment to oral compounds in Eisenmenger syndrome. *The European Respiratory Journal*.

[47] van Dissel AC, Post MC, Sieswerda GT, Vliegen HW, van Dijk AP, Mulder BJ (2021). Selexipag for pulmonary arterial hypertension in a wide range of adult congenital heart disease. *International Journal of Cardiology Congenital Heart Disease*.

[48] Hoeper MM, Al-Hiti H, Benza RL, Chang SA, Corris PA, Gibbs JSR (2021). Switching to riociguat versus maintenance therapy with phosphodiesterase-5 inhibitors in patients with pulmonary arterial hypertension (REPLACE): a multicentre, open-label, randomised controlled trial. *The Lancet. Respiratory Medicine*.

[49] Diller GP, Kempny A, Inuzuka R, Radke R, Wort SJ, Baumgartner H (2014). Survival prospects of treatment naïve patients with Eisenmenger: a systematic review of the literature and report of own experience. *Heart (British Cardiac Society)*.

[50] Broberg CS, Jayaweera AR, Diller GP, Prasad SK, Thein SL, Bax BE (2011). Seeking optimal relation between oxygen saturation and hemoglobin concentration in adults with cyanosis from congenital heart disease. *The American Journal of Cardiology*.

[51] Khairy P, Landzberg MJ, Gatzoulis MA, Mercier LA, Fernandes SM, Côté JM (2006). Transvenous pacing leads and systemic thromboemboli in patients with intracardiac shunts: a multicenter study. *Circulation*.

[52] Freisinger E, Gerß J, Makowski L, Marschall U, Reinecke H, Baumgartner H (2020). Current use and safety of novel oral anticoagulants in adults with congenital heart disease: results of a nationwide analysis including more than 44 000 patients. *European Heart Journal*.

[53] Galiè N, Saia F, Palazzini M, Manes A, Russo V, Bacchi Reggiani ML (2017). Left Main Coronary Artery Compression in Patients With Pulmonary Arterial Hypertension and Angina. *Journal of the American College of Cardiology*.

[54] Mesquita SMF, Castro CRP, Ikari NM, Oliveira SA, Lopes AA (2004). Likelihood of left main coronary artery compression based on pulmonary trunk diameter in patients with pulmonary hypertension. *The American Journal of Medicine*.

[55] Regitz-Zagrosek V, Roos-Hesselink JW, Bauersachs J, Blomström-Lundqvist C, Cífková R, De Bonis M (2018). 2018 ESC Guidelines for the management of cardiovascular diseases during pregnancy. *European Heart Journal*.

[56] Jaïs X, Olsson KM, Barbera JA, Blanco I, Torbicki A, Peacock A (2012). Pregnancy outcomes in pulmonary arterial hypertension in the modern management era. *The European Respiratory Journal*.

[57] Sertic F, Han J, Diagne D, Richards T, Chavez L, Berg A (2020). Not All Septal Defects Are Equal: Outcomes of Bilateral Lung Transplant With Cardiac Defect Repair vs Combined Heart-Lung Transplant in Patients With Eisenmenger Syndrome in the United States. *Chest*.

[58] Hjortshøj CS, Gilljam T, Dellgren G, Pentikäinen MO, Möller T, Jensen AS (2020). Outcome after heart-lung or lung transplantation in patients with Eisenmenger syndrome. *Heart (British Cardiac Society)*.

[59] Dimopoulos K, Inuzuka R, Goletto S, Giannakoulas G, Swan L, Wort SJ (2010). Improved survival among patients with Eisenmenger syndrome receiving advanced therapy for pulmonary arterial hypertension. *Circulation*.

